# Glutamine Supplementation: A Possible Strategy to Help Mitigate Health Risks of Heat-Related Illness

**DOI:** 10.1155/jnme/1638244

**Published:** 2024-11-22

**Authors:** Micah Zuhl, Jonathan Specht, Sage Beatty, Christine Mermier

**Affiliations:** ^1^Exercise Science Division, School of Health Sciences, Central Michigan University, Mount Pleasant, Michigan, USA; ^2^Department of Health, Exercise, and Sports Sciences, University of New Mexico, Albuquerque, New Mexico, USA

**Keywords:** exercise, glutamine, heat, illness, occupation

## Abstract

A rise in body temperature caused by physical work, including exercise, in a hot climate can lead to heat-related illnesses such as exertional heat exhaustion and stroke. Individuals who work physically demanding occupations in hot environments are at heightened risk of heat injury. The mechanisms that contribute to heat illness resulting from physical work in the heat are complex and include dehydration, tissue ischemia and damage, oxidative stress, and inflammatory events. Therefore, it is important to develop strategies that address these mechanistic underpinnings to prevent exacerbation to heat illness. Glutamine is an amino acid that has been considered conditionally essential during situations of biological stress (e.g., tissue burn, exercise, sepsis) due to high rates of tissue consumption. Evidence suggests that glutamine may serve as an important nutrient during heat stress and when combined with other preventative measures (e.g., cooling techniques, work/rest ratios, clothing) may help to mitigate heat illness among individuals working in extreme climates. The aim of this review is to examine the current literature on the role of glutamine during heat stress.

## 1. Introduction

Physical work in a hot environment can cause an increase in core temperature (hyperthermia), excessive fatigue, dehydration, and impaired cognitive performance [1]. These factors contribute to exertional heat illnesses which include exertional heat exhaustion (EHE); exertional heat injuries (EHI), such as gastrointestinal (GI), kidney, liver, or muscle damage as a result of hyperthermia; and more severe exertional heat stroke (EHS), i.e., a life-threatening condition characterized by hyperthermia along with central nervous system failure [2]. Exertional heat illnesses develop from external heat exposure combined with internal heat production (i.e., heavy physical work), which cause an increase in core temperature that is managed via the cardiovascular system's ability to increase blood flow to the skin surface for heat dissipation [1]. Myocardial strain is exacerbated by declining circulating volume (via sweating), and competition between exercising muscle and skin for blood delivery [3]. The re-direction of blood flow to the skin results in a reduced delivery to other tissue beds, resulting in relative hypovolemia. Various tissues such as such as brain, gut, and liver are often affected [4–7]. Prolonged sweat loss then causes a situation of absolute hypovolemia, and if severe, ischemia can arise causing tissue damage and production of circulating free radicals, inflammatory agents (e.g., cytokines), and endotoxins [4, 8–10]. The management of core temperature during heat stress (HS) is paramount to mitigating related illness and injury; however, the degree to which these downstream events occur (e.g., tissue ischemia, inflammation, and circulating molecules) during mild exertional hyperthermic events is not well known [11]. Evidence suggests that HS during prolonged exercise can cause both intestinal wall damage and further upregulate inflammatory markers [11]. In addition, the knowledge of daily work in the heat (e.g., construction laborers, agriculture workers) on markers of heat illness is also incomplete among humans. e.g., a high prevalence of impaired renal function was reported among sugarcane cutters, which was attributed to the physiological impact of daily work in a hot and humid environment [12].

The complexities of prolonged physical work in the heat present a challenge for developing and implementing prevention strategies. Novel intra-event cooling strategies such as water- and air-cooled vests, cold towels, menthol shirts, and ice packs have been shown to delay hyperthermia but a rise in core temperature eventually occurs [13, 14]. Hydration strategies have also been explored to help mitigate cardiovascular strain and tissue damage [15, 16]. Markedly less is known about the potential benefit of dietary supplements for mitigating heat illnesses [17]. Various antioxidants, dietary nitrates, amino acids, probiotics, and creatine have been tested among humans and animals on various physiological outcomes of HS [17]. The aims of these studies were to identify a supplement that can match the mechanistic underpinnings of heat illnesses (e.g., hydration, inflammation, oxidative stress), and when combined with cooling strategies, may provide a comprehensive approach for prevention.

Evidence suggests that glutamine is an ideal supplement to serve this role due to versatility of the amino acid in supporting tissue protection, immune function, and hydration [18, 19]. The aim of this review is to provide a critical exploration of glutamine's role in protecting humans against exertional heat illness. Included is an examination of mechanisms related to tissue damage, inflammation, and hydration, along with cellular and oxidative stress. To justify the importance of novel strategies to combat heat illnesses, a discussion regarding the prevalence and potential economic and mortality impacts will be highlighted as well. Throughout the review, the adopted term HS is used to identify physical work, including exercise, that leads to a rise in core temperature.

## 2. Prevalence of Exertional Heat Illnesses

Individuals working in physically demanding professions such as construction, agriculture, firefighting (both structure and wildland), police, warfighters, manufacturing, and warehousing are at heightened risk for EHI. Workers are four times more likely to experience heat injury (e.g., dehydration, palor, nausea, vomiting, confusion) after a single shift under hot conditions when compared to workers in ambient temperatures, and 15% of individuals who consistently work in the heat are susceptible to heat-related injuries [20]. Most vulnerable are individuals from lower-income groups, casual workers, construction laborers, those with preexisting health concerns, aging workers, and those with a history of heat illness [21–23]. The global estimates suggest that a fatal case of occupational HS is expected to occur every 14–24 min globally by 2030 [24]. The workforce productivity loss due to heat in the United States is projected to be 0.21%, which is equivalent to nearly 400,000 full-time jobs with an economic impact exceeding $2.4 trillion [21, 25].

Athletes and military service members are also at heightened risk for heat illness. Among competitive athletes, EHS, which is the most severe exertional heat illness, is a leading cause of sudden death [26, 27]. American football has historically had the highest rates of heat-related fatalities [28]. Determining the incidence of less severe EHIs such as heat injury or heat exhaustion among athletes has been much more difficult. Between 2005 and 2009, The U.S. Centers for Disease Control and Prevention (CDC) reported a weighted yearly average of 9237 cases of EHIs among high school athletes. The NCAA Injury Surveillance Program (166 institutions and 2048 team-seasons) received reports of only 232 exertional heat illnesses during sanctioned practices and competitions among collegiate athletes between 2009 and 2014 [29]. It's unknown if a lack of reporting exists, and cases of exertional heat illnesses during nonsanctioned practices are undetermined. Collegiate sports that put athletes at greatest risk were American football and soccer (both men and women). Rates among recreational athletes was reported to be nearly 6000 cases per year between 2001 and 2006, but current data do not exist [30]. There is a clear need to improve recording and reporting of nonfatal exertional heat illness occurrence among athletes at all levels; however, the immense challenge of such an endeavor is extraordinary.

Recent reports among U.S. Armed Service members indicated that in 2022, EHS and heat exhaustion occurred at rates of 32.1 and 147.7 per 100,000 person-years, respectively, with a total number of 12,228 cases between 2018 and 2022 [31]. At greatest risk were males and individuals between 20 and 29 years of age. Interestingly, only 3.1% of reported heat illnesses among military personnel occurred outside of the United Sates between 2018 and 2022. The U.S. military has been forthright in documenting and reporting cases since 2001; comparatively, detailed data from military forces across the globe is rarely published [32].

Organizations such as the National Institute for Occupational Safety and Health (NIOSH) and National Athletic Trainer's Association (NATA) have published recommendations for preventing and treating exertional heat illnesses [33, 34]. These have included adjusting work/exercise and rest cycles, reducing physical effort, enhancing heat tolerance via acclimation, worker and athlete training and recognition of symptoms, access to cool potable drinking water, and medical screening. A review of OSHA heat enforcement demonstrated that over 80% of employers did not rely on national standard approaches for heat injury mitigation [35]. Moreover, it was recently demonstrated that compliance with heat prevention guidelines was insufficient for reducing risk for heat illness among California farm workers [36]. This may indicate that further heat mitigation strategies are necessary.

## 3. Physiological Response to Heat Strain

Heat strain occurs when an imbalance of heat production and dissipation results in a rise in core temperature, which can be partially mitigated via the sweat response. Unless fluids are actively consumed to match fluid loss, a significant amount of water depletion (i.e., absolute hypovolemia) will occur, and can exacerbate many of the consequences of HS [37]. Dehydration paired with hyperthermia from HS leads to cardiac strain and increased blood viscosity, ultimately resulting in decreased systemic blood flow. A consequential response is relative hypovolemia from the shunting of blood away from splanchnic circulation to assist with core temperature control causing a milieu of reactive oxygen species (ROS) [38]. Oxidative stress within the intestinal epithelial membranes degrades the integrity of tight junction (TJ) proteins and facilitates endotoxin leakage into the portal circulation, and further release of inflammatory mediators (i.e., cytokines and chemokines) via circulating leukocytes (fig) [39].

**Figure 1:**
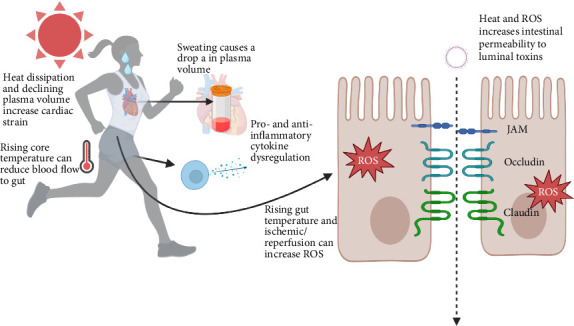
The central role of the gastrointestinal damage during physical work in the heat. Created with BioRender.com.

The central role of GI tract in EHIs was first hypothesized by Snipe et al., and further human experiments demonstrated the GI permeability–endotoxin–inflammation pathway after exercise in the heat [11, 40, 41]. Studies have elucidated that heat can induce alterations in the expression and localization of TJ proteins, such as occludin, claudins, and zonula occludens, leading to increased intestinal permeability [42, 43]. Compromised integrity of TJs permits paracellular passage of luminal antigens, toxins, and pathogens into the bloodstream (see Figure 1). GI insult is exacerbated during higher intensity bouts of exercise, and damage is also associated with the rise in the core temperature [44, 45]. Protecting the intestine during prolonged and intense HS is considered a central preventative measure against exertional heat illnesses [46]. Maintenance of upstream mechanisms such as hydration and preservation of circulating blood volume is another important preventative measure. Considerations must also be made regarding the inflammatory response that often follows GI insult and endotoxin leakage. Therefore, a treatment or supplement that helps preserve hydration, offers GI protection, and mediates the immune response would be ideal in the defense against exertional heat illnesses and specifically EHI. The following sections provide further detail into each mechanism and the role of glutamine.

## 4. Glutamine's Role in the Body During Physical Stress

Glutamine is the most abundant amino acid in the body and is central for supporting metabolic intermediary metabolism, ammonia transport, and pH balance [47–49]. Isotopic techniques have estimated that endogenous production is 40–80 g/day, and approximately 70–80 g are distributed in the whole body (dependent upon body composition) making up ∼20% of the circulating free amino acid pool [50, 51]. It is nutritionally classified as a nonessential amino acid; however, under conditions of trauma (e.g., severe burn, surgery), exhaustive physical exercise, and sepsis, tissue uptake and utilization of glutamine increases substantially [52, 53]. The rise in glutamine use during stress and catabolic conditions is met via intramuscular free glutamine and plasma levels, which both decline substantially under conditions of sepsis and physical trauma [54, 55]. Tissue uptake of glutamine during periods of stress supports metabolism, cellular defenses, molecular signaling, and glutathione (GSH) synthesis [51]. For example, a 50% decrease in postoperative plasma glutamine has been reported among surgical patients [56]. The intestinal tract, immune cells, and liver are believed to be major consumers of glutamine during periods of high stress [57, 58]. The interorgan and tissue flux support cellular integrity in the intestine; immune system proliferation and energy metabolism; and regulation of antioxidant defenses and nitrogen metabolism in the liver [59, 60]. The high rates of glutamine consumption during stress have led to the amino acid being considered conditionally essential [19].

Circulating glutamine levels appear to be stable during long duration (2 h) exercise but are shown to decrease by 16%–20% in recovery [61, 62]. Postexercise decline may be duration dependent with prolonged endurance bouts causing large drops in glutamine [63, 64]. For example, 140 min of cycling until exhaustion caused a 19% (682 to 552 *μ*mol^−1^) decrease in plasma glutamine that was detected after 2 h of recovery and returned to baseline by 7 h of recovery [65]. Limited evidence also suggests that high-intensity interval exercise lasting more than 30 min lowers recovery plasma glutamine [66]. The liver is a major consumer of plasma glutamine during periods of prolonged stress (e.g., physical exercise) where hepatic glutamine uptake has been shown to increase sixfold during exercise in dogs [67]. A large amount of glutamine uptake during exercise was used for nitrogen metabolism and urea production [68–70]. Hepatic glutamine is also a likely predominant gluconeogenic amino acid for blood glucose maintenance during prolonged exercise [71]. Glutamine use in the liver during exercise may further contribute to combatting oxidative stress via GSH synthesis through induction of the erythroid 2-related factor 2 (Nrf2) pathway [72, 73].

The gut and lymphocytes are also avid consumers of glutamine during physical stress such as exercise. For example, Krishna et al. demonstrated a two- to threefold increase in intestinal glutamine extraction during exercise in dogs [67]. Glutamine's actions in intestinal epithelial cells during stress are to support enterocyte metabolism, dietary nitrogen and carbon processing, cell proliferation, and gut barrier maintenance [74–77]. The GI tract is considered the principal consumer of glutamine with evidence suggesting that > 50% of enterally administered glutamine is metabolized within the intestinal mucosa [78]. Glutamine may be a more efficient energy fuel for enterocytes as evidence shows a yield of 9 ATP/1 mol glutamine vs. 8 ATP/1 mol glucose [79]. The nonaerobic glycolytic pathway supplies the bulk of the ATP for enterocytes, and glutamine utilization may spare both endogenous and exogenous glucose substrates to support other tissues [75, 79–81].

Glutamine is an important biosynthetic and metabolic nutrient for both T and B lymphocytes, macrophages, and peripheral blood mononuclear cells during prolonged exercise and other situations of physical stress [82]. These cells all demonstrate high rates of glutaminase activity, and accelerated rates of glutamine utilization have been reported to be greater than glucose uptake [82, 83]. Through the process of glutaminolysis, glutamine is converted to glutamate, asparate, and alanine to support cellular metabolism and is essential for both T and B cell regulatory function [84, 85]. The oxidation of glutamine for energy generation within lymphocytes and macrophages is incomplete which indicates that the amino acid may be important for other functional roles within immune cells. Specifically, glutamine can be used to support the synthesis of purines and pyridines, along with induction of protein transcription factors that regulate cellular proliferation (c-jun N-terminal kinases, JNK, and activator protein-1, AP-1) and cellular repair (heat shock proteins, Hsps) [47, 86]. Glutamine and glutamate can also be used to generate essential compounds in neutrophils for defense against ROS [87]. Glutamine may also regulate both pro- and anti-inflammatory cytokine signaling in various immune cells under conditions of illness and physical stress such as extreme exercise. Glutamine consumption by leukocytes helps to explain skeletal muscle glutamine depletion during situations of bodily stress. Glutamine is also used to synthesize antioxidant defenses in red blood cells and has emerged as an important nutrient to prevent complications of sickle cell disease [88].

**Figure 2:**
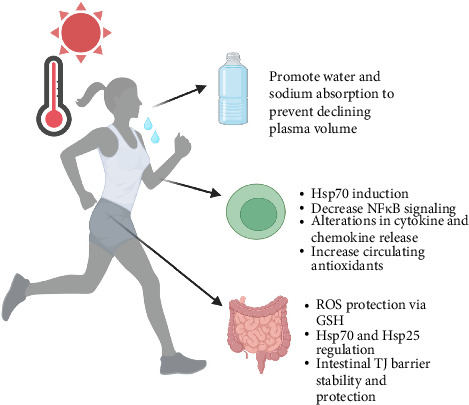
The role of glutamine during physical work in the heat. Evidence suggests that glutamine ingestion can promote hydration, regulate cellular defenses through Hsp70 induction and regulation of inflammatory mediators, along with stabilizing the intestinal epithelial wall. Created with BioRender.com.

Shifts in glutamine tissue transport and utilization during HS is not well understood in humans. Sixty minutes of treadmill running at 70% intensity in a heated environment (30°C) caused a small nonsignificant decrease in circulating glutamine levels at immediately after, 2 h, and 4 h postexercise time points [9, 89, 90]. Levels decreased significantly after 87 min of exercise in a markedly hotter environment (38°C) that included various activities such as treadmill walking, resistance exercises, and box stepping [91]. It is clear that an increase in plasma glutamine levels through oral supplementation results in a substantial decline in glutamine at post-HS time points. For example, in numerous studies, our group and others have reported a significant increase in glutamine levels after supplementation followed by a decline after HS that can be further rescued with continued glutamine ingestion [9, 89–92]. The sharp decrease after HS may indicate glutamine tissue uptake and utilization in various biochemical reactions. The following sections highlight evidence of glutamine's role in these systems during HS and other related stressors.  Figure 2 provides a summary.

### 4.1. Intestinal Damage/Dysfunction During Heat Stress

The role of glutamine in protecting the gut during heat exposure is best identified by examining glutamine supplementation trials. Animal and intestinal cell culture studies have shown protective effects of glutamine supplementation during heat exposure interventions [93–95]. Soares et al. reported improved intestinal barrier function and reduced bacterial translocation in mice that were fed a glutamine-supplemented diet versus isoenergetic and isonitrogenous placebo chow for 7 days prior to passive HS [96]. Lower levels of plasma endotoxin and increased survival following classic heat stroke were demonstrated in rats after 5 days of glutamine ingestion compared to control [97]. Heightened resistance to injury and apoptosis has been further shown in glutamine supplemented intestinal cell cultures after lethal heat insult [98, 99].

Our group has reported improved intestinal barrier function and lower damage after oral glutamine ingestion among humans exercising in the heat. The rise in intestinal permeability was abolished after 60 min of high-intensity treadmill running when high-dose (0.90 g/kg/day) glutamine was ingested for 7 days prior (chronic supplementation) or immediately before (1 single dose) [9, 89]. Pugh et al. tested the effect of glutamine dose (0.00, 0.25, 0.50, 0.90 g/kg/day) on intestinal permeability among humans after 1 h of treadmill exercise in the heat, and found that glutamine attenuated permeability in a dose-dependent fashion [90]. Acute glutamine ingestion (dosed at 0.30 g/kg/day) prior to 87 min of HS also led to reduced intestinal damage, identified by plasma fatty acid-binding protein (IFABP), that was observed 24 h after HS [91, 100]. Circulating IFABP levels after a 20-km time trial in the heat were lower among cyclists who ingested a glutamine bolus (0.90 g/kg) versus a placebo prior to exercise. Conversely, acute low-dose oral glutamine (0.30 g/kg/d) ingestion had no effect on intestinal permeability and IFABP changes after exhaustive running (∼22 min) in heat and after 80 min of treadmill walking in the heat [101, 102]. The lack of consistent findings may be explained by the measured time points as intestinal permeability was captured 1 h postexercise compared to 5 h postexercise in previous studies. Plasma IFABP was also collected at the postexercise time point only, versus 2-, 4-, and 24-h postexercise in similar trials [91]. Another explanation is that the HS was not severe enough to cause intestinal disruption [45]. However, the null findings may also suggest that a higher dose of glutamine is required before HS and that benefits favor chronic supplementation over a single dose.

Several mechanisms explain the protective benefits of glutamine on intestinal barrier stability and function during HS. First, glutamine has been shown to activate heat shock factor-1 (Hsf-1), the transcription factor for inducible heat shock proteins or Hsps (namely Hsp70/72 and Hsp25) [99, 103]. The heat shock response is a cytoprotective event that promotes cell survival through protein re-folding and trafficking combined with modulation of inflammatory events via regulation of the NF*κ*B pathway [104, 105]. Mild stress causes an increase in Hsp70 and Hsp25 levels in intestinal epithelial cells designed to maintain cellular structure and function. Upon prolonged or more severe stress, inflammatory mediators such as TNF-*α* and IFN-*γ* increase and downregulate Hsp70 and 25 [106, 107]. Evidence suggests that glutamine is necessary for Hsp70 and Hsp25 stabilization in intestinal cells during HS where glutamine inhibition diminishes Hsp induction and cellular protection [99, 103]. Glutamine promotes Hsf-1, Hsp70, and Hsp25 gene induction through the hexosamine biosynthetic pathway (HBP), which splits from glycolysis at the fructose-6-phosphate step [108, 109]. HBP metabolism of glutamine leads to *O*-glycosylation (via *O*-linked *N*-acetylglucosamine or GlcNAc), nuclear translocation, and transcriptional regulation of Hsf-1 and Sp-1 and thus Hsp70 and Hsp25 synthesis [108, 110]. The O-GlcNAc transferase (OGT) enzyme catalyzes GlcNAcylation of proteins in mammals, and OGT silencing prevented glutamine linked *O*-glycosylation and activation of Hsf-1 and Hsp70 in heat stressed mouse fibroblasts [108, 111].

Heat shock proteins protect the gut during HS by stabilizing intestinal TJ proteins. In a Caco-2 cell model, Dokladny, Moseley, and Ma demonstrated that Hsp70 was responsible for stabilization of occludin during physiologically relevant thermal insult [112]. Hsp70 may exert an effect on occludin by physical binding and stabilization, which has been shown to occur during heat exposure [113]. Hsf-1 and Hsp70 induction was also linked to zona occludens and claudin-1 mRNA expression in rat jejunum after HS [114]. Occludin, claudins, and zona occludens (ZO-1, ZO-2, and ZO-3) are key proteins that regulate the TJ barrier and paracellular transport in the gut. Another mechanism for Hsp protection in the gut is by regulating the state of intestinal epithelial actomyosin contraction. The TJ is influenced by the contractile state of the actin-myosin cytoskeleton within adjacent intestinal epithelial cells [115]. High temperature environments can cause phosphorylation of myosin light chain kinases (MLCK) via protein kinase C (PKC) with increased permeability as a result [116]. Pretreatment of T84 cells (human colon-derived crypt-like cell line) with Hsp70 effectively inhibited PKC phosphorylation of MLCK during HS [116]. In summary, Hsps prevent intestinal permeability through physical stabilization and protection of TJ proteins along with regulating contraction of the actomyosin chain. Jejunal enterocytes supplemented with glutamine had increased transepithelial electrical resistance (TEER) in accordance with occludin, claudins, and ZOs expression, which may be linked to glutamine regulation of the Hsp family [117].

Another mechanism that may explain glutamine protection in the gut is through the control of mitogen-activated protein kinases (MAPK) and phosphatidylinositol 3-kinase (PI3K)/AKT pathways. Key MAPKs are extracellular signal-related kinase (ERK), JNK, and p38 MAPK [118]. ERK signals cell proliferation, DNA synthesis, and promotes anti-apoptosis while JNK and p38 are stress related and lead to cell death in many cell lines, including the intestinal epithelial cells [119]. Larson et al. demonstrated that glutamine mediates cell survival through ERK phosphorylation and activation [119].

A third mechanism for glutamine intestinal benefits is through the control of inflammatory mediators in the gut during various forms of physical stress. Hou et al. reported decreased colonic expression of the NF*κ*B inflammatory pathway in mice supplemented with glutamine prior to chemical insult [120]. In intestinal epithelial cells, NF*κ*B is a transcription factor that controls inflammation, innate immunity, and tissue damage [121]. NF*κ*B is often considered a double-edge sword where on one hand, gene silencing exacerbates tissue damage and inflammation and on the other hand, overexpression of NF*κ*B does the same [85, 122]. This is evident in many chronic inflammatory disorders such as inflammatory bowel disease (IBD), rheumatoid arthritis, and coronary artery disease [123]. Unstimulated NF*κ*B is physically bound in the cytosol by the I*κ*B kinase (IKK) complex consisting of I*κ*B*α* and I*κ*B*β* subunits [124, 125]. Activation results in phosphorylation and degradation of I*κ*B*α*, followed by nuclear translocation of NF*κ*B into the nucleus for the transcription of numerous genes, including cytokines (e.g., IL-1, IL-6, IL-8, TNF), chemokines (e.g., MCP-1), adhesion molecules (e.g., ICAM-1), and immune cell activity [126, 127]. Oxidative, viral, and microbial cytokine stressors along with protein signaling such as PKC and MAPK are known activators of NF*κ*B [125, 128]. While unclear in human studies, thermal insult has been shown to induce intestinal NF*κ*B activation in animal studies [129]. For example, Xia et al. reported heightened NF*κ*B activity in intestinal tissue of heat stroke mice that were associated with elevations in circulating TNF-*α*, IL-6, and IL-1*β* [130]. Similar findings were presented in the ileum mucosa of pigs after 7 days of chronic HS [131]. Glutamine may regulate NF*κ*B activation by decreasing MAPK and PKC signaling, which was observed in colonic tissue of individuals with Crohns supplemented with glutamine [132, 133]. Supplementation also prevented I*κ*B*α* degradation, NF*κ*B induction, and downstream cytokine synthesis during inflammatory insult in human intestinal cells, indicating another site of glutamine control [134]. Glutamine upregulation of Hsp70 has been shown to physically bind to I*κ*B*α* and prevent ubiquitination, which likely explains the mechanism of glutamine stabilization of I*κ*B*α* [134, 135].

Glutamine also controls inflammation in the gut by modulating free radicals that can be generated from the ischemic-reperfusion cycling that occurs during hyperthermia [136]. ROS production generated from oxidative stress has been proposed as a heat illness-related pathology [136]. In this model, blood flow is re-directed away from the gut to support thermoregulation, resulting in cellular hypoxia, production of ROS, and tissue damage [136]. Daily HS was shown to increase production of ROS and decrease antioxidant activity in rat intestinal epithelial cells but treatment with glutamine protected the cells against both heat and oxidative damage [98, 137]. Glutamine serves as a precursor for GSH synthesis, which is a central cellular antioxidant [52]. Intracellular levels of GSH were increased in the intestine, liver, lung, and muscle after glutamine ingestion [138]. In the gut specifically, Cao et al. demonstrated that glutamine metabolism provided glutamate to synthesize GSH in rat intestinal tissue, and glutamine fed animals had a threefold higher expression of GSH [139]. Glutamine also preserved GSH levels during intestinal ischemic reperfusion insult in animals [140]. The ratio of reduced (GSH) and oxidized (GSSG) intracellular GSH determines the redox potential, and ROS production and scavenging by GSH during ischemic events lowers the ratio. Intestinal antioxidant defenses are dependent upon maintaining a high cellular concentration of GSH in proportion to GSSG, and glutamine serves as a precursor to support maintenance of this ratio [141].

In summary, glutamine protects the gut during the HS by stabilizing the TJ barrier via preservation of inducible heat shock proteins (Hsp70 and Hsp25), and through control of the intestinal epithelial cell cytoskeleton. Glutamine modulates the inflammatory response that accompanies prolonged HS via control of the NF*κ*B and GSH pathways.

### 4.2. Immune Support During Heat Stress

The immune response during classic heatstroke (i.e., passive heat exposure) has been characterized by elevated levels of inflammatory cytokines that have been monitored at the time of collapse or upon cooling. Bouchama et al. monitored pyrogenic cytokine levels among 28 classic heatstroke patients and reported elevated IL-6, IL-1*β*, and INF-*γ* in all patients, and IL-6 concentrations were associated with severity of heat illness [142]. Similarly, elevated levels of IL-6 were detected upon hospital admission among EHS patients [143]. A correlation between inflammatory cytokine activity and core temperature changes during heatstroke has been challenging due to the complexity between pro- and anti-inflammatory signaling. For example, Hashim et al. observed elevations in anti-inflammatory cytokines, IL-1ra and TNFs r-II, among exertional heatstroke patients, which inhibit IL-1 (*β* and *α*) and TNF-*α*, respectively [143–145]. Higher levels of TNFsr-II were associated with mortality. Moreover, classic heatstroke in a baboon model was characterized by a rise in anti-inflammatory cytokine, IL-10 and chemokine, IL-8 along with IL-1ra and TNFs r-II that were observed early (∼1 h) into the heat insult but mortality followed [146]. This reveals that an exaggerated anti-inflammatory cytokine response may worsen heatstroke outcomes, which is further demonstrated by IL-6 and TNF-*α* knockout mice showing higher classic heat stroke-related mortality [147]. Together, these findings indicate that severe heat illness causes dysregulation of anti-inflammatory modulators and likely play a role in the pathology of both exertional and classic forms of heatstroke. In the only known study examining glutamine supplementation on cytokine release after classic heatstroke, it was recently reported that 7 days of glutamine supplementation lowered IL-8 and mitigated the rise in IL-6 levels among rats after heatstroke compared to placebo control indicating a modulation of inflammatory cytokines, but the changes in anti-inflammatory cytokines were not reported [148].

The inflammatory response to nonlife threatening HS and the impact on systemic function is not well defined. Snipe et al. reported higher peak IL-6, IL-10, and IL-1ra values among healthy adults after 120 min of running in the heat compared to a nonheat running control [11]. The research group also explored anti-inflammatory to pro-inflammatory ratios measured as IL1*β*:IL-10 and TNF-*α*:IL-10 ratios and reported lower ratios throughout the HS trial [11]. These findings align with heightened anti-inflammatory responses during EHS noted above. Interestingly, Hailes et al. observed heightened basal levels of several anti-inflammatory agents such as IL-10, IL-1ra, and TNFsr-II after four consecutive days of HS, and suggested a modulated inflammatory profile that may alter the immune response to additional heat exposures [149]. Conversely, pro-inflammatory agents such as TNF-*α*, IL-1*β*, IL-12p40, and MCP-1 have been shown to increase after exercise in the heat [41, 149, 150]. Circulating cortisol and LPS are possible mediators of the inflammatory response as both increase after HS compared to exercise control [11]. While the pro- and anti-inflammatory responses to HS are complex, it's clear that prolonged heat exposure can cause dysregulation of this system, and put one at risk for exertional heat illnesses, especially among individuals who are unacclimated [149, 150].

Like glutamine's role in intestinal tissue during HS, the effect of the amino acid on immune function during HS is multifactorial. Neutrophils, macrophages, and lymphocytes are all large consumers of glutamine, and utilization rates rise during cellular stress to support metabolic activity and promote protein signaling pathways that influence cellular defenses along with cell proliferation and cytokine production [18, 47]. Zheng, Chen, and Zhou monitored all circulating immune cell types after exhaustive HS among untrained males who consumed 0.6 g/kg of glutamine 30 min prior to exercise [151]. Only T cell lymphocyte mobilization into the peripheral blood was higher in the glutamine trial compared to HS alone [151]. Namely, the responsiveness of CD3+ and CD3+CD8+ T lymphocytes was observed, which regulates the activities of T helper and cytotoxic T naïve cells. Cell model experiments have demonstrated that glutamine is essential for T lymphocyte proliferation [152]. This may indicate heightened immune surveillance to invading pathogens. In animal models, consecutive days of heat exposure led to a depression in CD3+ and CD8+ T cells in broiler chickens [153]. T lymphocytes are capable of releasing pro-inflammatory cytokines such as IL-6, IL-2, IL-1, and TNF-*α*; however, cytokine levels were unchanged immediately after the glutamine supplemented HS trial and explained by the low intensity (40%) and short duration (<40 min) of the trial. High-dose glutamine (0.90 g/kg) ingested prior to 60 min of high intensity HS (70%) led to lower TNF-*α* levels compared to placebo trial that was detected at 4 h into recovery [89]. Unfortunately, little is known about the cytokine and chemokine response to HS after glutamine supplementation. However, these findings may help to better focus researchers on T cell function and the role of glutamine [151].

Glutamine regulation of heat shock response also plays a role in circulating immune cells during HS. Both acute and 7 days of high dose (0.90 g/kg) prior to exercise in the heat increased Hsp70 expression in PBMCs observed at 2- and 4-h postexercise [89, 154]. Glutamine dosed at 0.30 g/kg and administered before and after consecutive days of prolonged HS increased PBMC Hsp70 levels after the second day [91]. In a similar trial, 0.15 g/kg of glutamine (dosed before and after HS) increased Hsp70 expression 4 h after HS on the first day compared to a placebo supplement [92]. As reviewed in intestinal epithelial cells, glutamine induction of Hsp70 may insert regulatory control of the NF*κ*B pathway through I*κ*B*α* stabilization and reduction in inflammatory cytokine release from PBMCs [89, 91, 92, 155]. However, the effect of glutamine combined with HS on Hsp70 activation, I*κ*B*α* preservation, and NF*κ*B inhibition in PBMCs have all been associations, and gene silencing models do not existing. Therefore, it's unknown if these findings are cause and effect or mediated through other regulatory pathways. However, glutamine's attenuation of NF*κ*B was shown to be dependent on Hsp70 activation in lung tissue from an animal sepsis model [97].

Glutamine may also play a role in antioxidant maintenance during HS, but limited human studies exist. Tissues other than the intestines are vulnerable to oxidative stress during prolonged heat exposure. Evidence has suggested that myocytes, skeletal muscle tissue, neurons, erythrocytes, and macrophages produce ROS during heat exposure [156]. Mitochondria are the primary source as ROS are the by-products of partially reduced oxygen that occurs during high rates of oxidative phosphorylation that can occur during heat exposure [156–158]. Key antioxidant systems that mitigate mitochondrial ROS include GSH, catalase, and superoxide dismutase [156]. Glutamine supplementation in broiler chickens exposed to HS resulted in higher levels of all three mentioned antioxidants regardless of tissue type [159]. In the only known human study, low-dose glutamine supplemented at 0.15 g/kg increased plasma total antioxidant capacity (TAC) 4 h after 78 min of HS [92]. The measure of TAC is used as an indirect measurement of systemic antioxidant levels and monitors the effects of antioxidant supplementation [160]. HS may exacerbate disorders linked to ROS, and chronic heat exposure may accelerate the negative impact of these molecules. Therefore, more work is needed to better understand glutamine's role in tissue antioxidant defenses during HS.

### 4.3. Hydration

Body water maintenance is pivotal to sustaining thermoregulation during long duration physical work in the heat. Dehydration by more than 2% can impair cognitive tasks such as attention and executive function which are essential in any work environment [161]. The impact of water loss on physical performance is not clear with declines in the range of 2%–4% linked to performance decrements in long duration activities [162]. Rehydration solutions and sports drinks often contain high amounts of sodium and glucose to promote water absorption and electrolyte replacement. American football athletes lost 2.5 g/h of sodium during a 4.5-h practice session (WBGT 25.9°C), and outdoor workers' (WBGT 29.3) loss ranged from 4.6 to 6 g over an 8- to 12-h work shift [163, 164]. These findings indicate that sodium replacement is important for individuals exposed to extreme physical and environmental challenges. Sodium ingested either within a hydration drink or in food increased fluid retention and restoration of plasma volume after a 2.5% body weight loss [165]. The addition of glucose promotes sodium absorption, glucose uptake, and water transport through the sodium-glucose co-transporter-1 (SGLT-1) channel [166, 167]. The linkage of glucose with obesity and diabetes has created some controversy regarding the value of the ingredient in beverage formulas. Evidence suggests that glutamine stimulates sodium and water absorption from the human jejunum, and may be considered as a substitute for glucose [168]. Coeffier et al. reported an increase in both sodium and water absorption after an infusion of a glutamine-enriched hydration mix among healthy adults [168]. Intestinal fluid and electrolyte uptake was maintained even in the face of induced secretory diarrhea [168]. Similarly, an L-alanyl-L-glutamine dipeptide infused drink was shown to increase sodium and water absorption along with improving physical and cognitive performance among males [169, 170]. The advantages of a glutamine beverage are effective hydration combined with intestinal and immune benefits that offers a multifaceted support against HS.

### 4.4. Fatigue

Fatigue is the failure of physical performance that results from both peripheral (i.e., skeletal muscle) and central (i.e., central nervous system) factors. Peripheral fatigue has been attributed to depletion of energy substrates, ammonia build-up, oxidative stress, and muscle proton build-up [171, 172]. Reduced neural motor drive along with changes in neurotransmitter such as increased serotonin and lowered dopamine are considered central fatigue mechanisms [171, 173]. Fatigue can also contribute to a decline in cognitive function that can occur during prolonged exercise or physical labor in a warm climate [174, 175]. Glutamine supplementation prior to prolonged exercise has improved markers of fatigue, but these findings have not been associated with improved physical performance [92, 176]. Regarding peripheral fatigue, glutamine serves as a major gluconeogenic amino acid in the liver and kidneys to support substrate metabolism during prolonged exercise [71]. Glutamine's role in energy metabolism prevents ammonia release, which occurs during amino acid oxidation in response to glycogen depletion during exercise [177]. Ammonia build-up during exercise is also attenuated because glutamine is the main nitrogen transporter and assists with ammonia removal. Bassini-Cameron et al. reported lower circulating ammonia in response to intense exercise among professional football players after chronic glutamine supplementation [177].

The impact of glutamine on mechanisms linked to central fatigue is less clear. It's unlikely that glutamine supplementation affects neural transmission since the blood brain barrier tightly regulates glutamine transport. Interestingly, evidence suggests that these transporters work by removing glutamine from the brain [19, 178]. Glutamine may work indirectly via the gut-brain axis, whereas the benefits of glutamine supplementation in the GI tract may contribute to changes in the central nervous system that improves cognitive function [179]. However, this is speculative as no studies have explored the effect of glutamine supplementation on cognition via the gut-brain axis. Other research also points toward glutamine supplementation having a positive effect on cognition, mood, and fatigue via the mechanisms highlighted in the previous sections, such as hydration, inflammation, and oxidative stress [180].

Several studies have shown that glutamine is an anti-fatigue amino acid, but limited data suggest improved physical performance as a result [176]. The mechanisms are likely linked to energy metabolism and ammonia regulation. Continued work is needed to better understand the influence of glutamine on fatigue in hot environments.

## 5. Glutamine Supplementation

The goal of glutamine supplementation is to increase circulating glutamine levels, which indirectly indicates a rise in intestinal glutamine uptake since > 50% of orally ingested glutamine is metabolized in the gut [78]. Glutamine dosed in the range of 0.15–0.90 g/kg/day increased plasma levels prior to HS [9, 90, 92]. Acute dosing at or above 0.90 has been linked to GI discomfort (e.g., bloating, diarrhea), which may indicate that an ideal dose-response exists [102]. Nava et al. administered 0.15 g/kg before and after HS (for a total of 0.30 g/day) for consecutive days (two HS trials) and observed a significant rise in pre-HS on day 1 (∼23.80 mg/dL) and on day 2 (∼28.97 mg/dL). Comparatively, a 0.90 g single bolus taken 2 h before HS increased (∼23.82 mg/dL) to a similar level [91]. These results show that multiple small doses were effective in increasing glutamine levels compared to a single large dose. In addition, dosing before and after HS may prevent a decrease in circulating glutamine levels that is often observed during recovery from prolonged and intense exercise [65, 91].

Acute supplementation versus chronic glutamine ingestion on circulating glutamine levels in humans is not clear. Seven days of dosing at 0.90 g/kg split into three doses per day (0.30 g/kg/dose) increased glutamine levels prior to HS similar to a single 0.90 g/kg dose [9, 89]. Fourteen days of glutamine supplementation at 0.40 g/day had no effect on circulating glutamine levels among healthy adults who performed nine consecutive days of interval training. However, it was unclear when blood samples were collected (before or after training sessions or glutamine ingestion) as glutamine levels have shown to shift after heavy exercise in supplementation trials [89, 181]. These findings inform that physical activity patterns have a huge impact on circulating glutamine levels during supplementation cycles. In summary, acute glutamine ingestion may be ideal prior to a single bout of exercise or HS, and if an individual is chronically exposed, then continued glutamine ingestion may be helpful. The impact of chronic supplementation with and without daily physical work on markers of HS exposure is needed. In addition, it's unknown if daily glutamine ingestion improves one's ability to acclimate to environmental heat exposure. What we do know is that an acute dose can increase glutamine levels substantially and higher circulating levels may improve HS outcomes.

Based on the thorough review of the glutamine and HS literature, we suggest the following dosing recommendations for optimal glutamine benefits: (1) Total daily glutamine supplementation should be between 0.15 and 0.30 g/kg/d (10.5–21 g for 70 kg individual), as these amounts increased glutamine levels without adverse effects [90, 91]; (2) For acute supplementation approaches, we recommend separating the amount into several smaller doses and ingesting before and after HS, e.g., consuming 0.075 g/kg/serving before and after HS increased and stabilized plasma glutamine levels [92]. A large single bolus should be avoided to prevent GI disturbance; and (3) Chronic supplementation strategies should follow similar guidelines with the daily dose split into multiple smaller quantities. Seven-day cycles or less appear to be optimal for raising glutamine levels prior to HS. The impact of longer supplementation routines is currently not known.

## 6. Conclusions

The forecasted occurrences of heat injury and death related to HS among those working in the heat is startling and agencies such as the U.S. Occupational Safety and Health Administration, International Labor Organization, and American Conference of Governmental Hygienists have raised alarms and published prevention strategies [33, 182]. The primary goal is to prevent a severe rise in core temperature through proper hydration and active cooling behaviors (e.g., work/rest cycles, appropriate clothing, cooling technologies). Functional nutritional aids offer another possible strategy to partner with prevention strategies. Glutamine is an ideal supplement to serve this function as it's been shown to address many of the mechanistic underpinnings of HS-related illness such as oxidative stress, GI damage, and inflammatory dysregulation. Benefits have been reported after supplementation with a mild dose of glutamine before and after HS. Continued research is needed in humans to better understand important inflammatory regulators during and after HS and the role of nutraceuticals, including glutamine.

## Data Availability

Data sharing is not applicable to this article as no new data were created or analyzed in this study.

## References

[1] Armstrong L. E., Casa D. J., Millard-Stafford M., Moran D. S., Pyne S. W., Roberts W. O. (2007). Exertional Heat Illness During Training and Competition. *Medicine & Science in Sports & Exercise*.

[2] Roberts W. O., Armstrong L. E., Sawka M. N., Yeargin S. W., Heled Y., O’Connor F. G. (2023). ACSM Expert Consensus Statement on Exertional Heat Illness: Recognition, Management, and Return to Activity. *Current Sports Medicine Reports*.

[3] Lucas R. A., Epstein Y., Kjellstrom T. (2014). Excessive Occupational Heat Exposure: A Significant Ergonomic Challenge and Health Risk for Current and Future Workers. *Extreme Physiology & Medicine*.

[4] Leon L. R., Helwig B. G. (2010). Heat Stroke: Role of the Systemic Inflammatory Response. *Journal of Applied Physiology*.

[5] Nielsen B., Nybo L. (2003). Cerebral Changes During Exercise in the Heat. *Sports Medicine*.

[6] Ho C., Beard J., Farrell P., Minson C., Kenney W. (1997). Age, Fitness, and Regional Blood Flow During Exercise in the Heat. *Journal of Applied Physiology*.

[7] Gaskell S. K., Burgell R., Wiklendt L., Dinning P., Costa R. J. (2022). Does Exertional Heat Stress Impact Gastrointestinal Function and Symptoms?. *Journal of Science and Medicine in Sport*.

[8] Kaldur T., Kals J., Ööpik V. (2014). Effects of Heat Acclimation on Changes in Oxidative Stress and Inflammation Caused by Endurance Capacity Test in the Heat. *Oxidative Medicine and Cellular Longevity*.

[9] Zuhl M. N., Lanphere K. R., Kravitz L. (2014). Effects of Oral Glutamine Supplementation on Exercise-Induced Gastrointestinal Permeability and Tight Junction Protein Expression. *Journal of Applied Physiology*.

[10] Lee B. J., Flood T. R., Galan-Lopez N. (2023). Changes in Surrogate Markers of Intestinal Epithelial Injury and Microbial Translocation in Young and Older Men During Prolonged Occupational Heat Stress in Temperate and Hot Conditions. *European Journal of Applied Physiology*.

[11] Snipe R. M., Khoo A., Kitic C. M., Gibson P. R., Costa R. J. (2018). The Impact of Exertional-Heat Stress on Gastrointestinal Integrity, Gastrointestinal Symptoms, Systemic Endotoxin and Cytokine Profile. *European Journal of Applied Physiology*.

[12] García-Trabanino R., Jarquín E., Wesseling C. (2015). Heat Stress, Dehydration, and Kidney Function in Sugarcane Cutters in El Salvador–a Cross-Shift Study of Workers at Risk of Mesoamerican Nephropathy. *Environmental Research*.

[13] Bishop P. A., Nunneley S. A., Constable S. H. (1991). Comparisons of Air and Liquid Personal Cooling for Intermittent Heavy Work in Moderate Temperatures. *American Industrial Hygiene Association Journal*.

[14] Keen M. L., Miller K. C., Zuhl M. N. (2017). Thermoregulatory and Perceptual Effects of a Percooling Garment Worn underneath an American Football Uniform. *The Journal of Strength & Conditioning Research*.

[15] Gonzalez-Alonso J., Heaps C., Coyle E. (1992). Rehydration After Exercise With Common Beverages and Water. *Int J Sports Med*.

[16] Chapman C. L., Johnson B. D., Vargas N. T., Hostler D., Parker M. D., Schlader Z. J. (2020). Both Hyperthermia and Dehydration during Physical Work in the Heat Contribute to the Risk of Acute Kidney Injury. *Journal of Applied Physiology*.

[17] Lee J. K., Tan B., Ogden H. B., Chapman S., Sawka M. N. (2022). Exertional Heat Stroke: Nutritional Considerations. *Experimental Physiology*.

[18] Cruzat V., Macedo Rogero M., Noel Keane K., Curi R., Newsholme P. (2018). Glutamine: Metabolism and Immune Function, Supplementation and Clinical Translation. *Nutrients*.

[19] Newsholme P., Diniz V. L. S., Dodd G. T., Cruzat V. (2023). Glutamine Metabolism and Optimal Immune and CNS Function. *Proceedings of the Nutrition Society*.

[20] Flouris A. D., Dinas P. C., Ioannou L. G (2018). Workers’ Health and Productivity under Occupational Heat Strain: a Systematic Review and Meta-Analysis. *The Lancet Planetary Health*.

[21] Szewczyk W., Mongelli I., Ciscar J.-C. (2021). Heat Stress, Labour Productivity and Adaptation in Europe—A Regional and Occupational Analysis. *Environmental Research Letters*.

[22] Ioannou L. G., Mantzios K., Tsoutsoubi L (2021). Occupational Heat Stress: Multi-Country Observations and Interventions. *International Journal of Environmental Research and Public Health*.

[23] Gubernot D. M., Anderson G. B., Hunting K. L. (2015). Characterizing Occupational Heat‐related Mortality in the United States, 2000–2010: An Analysis Using the Census of Fatal Occupational Injuries Database. *American Journal of Industrial Medicine*.

[24] Kjellstrom T., Freyberg C., Lemke B., Otto M., Briggs D. (2018). Estimating Population Heat Exposure and Impacts on Working People in Conjunction With Climate Change. *International Journal of Biometeorology*.

[25] Morrissey M. C., Brewer G. J., Williams W. J., Quinn T., Casa D. J. (2021). Impact of Occupational Heat Stress on Worker Productivity and Economic Cost. *American Journal of Industrial Medicine*.

[26] Yankelson L., Sadeh B., Gershovitz L. (2014). Life-threatening Events During Endurance Sports: Is Heat Stroke More Prevalent Than Arrhythmic Death?. *Journal of the American College of Cardiology*.

[27] Maron B. J., Doerer J. J., Haas T. S., Tierney D. M., Mueller F. O. (2009). Sudden Deaths in Young Competitive Athletes: Analysis of 1866 Deaths in the United States, 1980–2006. *Circulation*.

[28] Grundstein A. J., Hosokawa Y., Casa D. J. (2018). Fatal Exertional Heat Stroke and American Football Players: the Need for Regional Heat-Safety Guidelines. *Journal of Athletic Training*.

[29] Yeargin S. W., Dompier T. P., Casa D. J., Hirschhorn R. M., Kerr Z. Y. (2019). Epidemiology of Exertional Heat Illnesses in National Collegiate Athletic Association Athletes During the 2009–2010 through 2014–2015 Academic Years. *Journal of Athletic Training*.

[30] Nelson N. G., Collins C. L., Comstock R. D., McKenzie L. B. (2011). Exertional Heat-Related Injuries Treated in Emergency Departments in the US, 1997–2006. *American Journal of Preventive Medicine*.

[31] DeGroot D., Henderson K., O’Connor F. (2022). Exertional Heat Illness at Fort Benning, GA: Unique Insights From the Army Heat Center. *Inside MS*.

[32] Alele F. O., Malau-Aduli B. S., Malau-Aduli A. E., J Crowe M. (2020). Epidemiology of Exertional Heat Illness in the Military: A Systematic Review of Observational Studies. *International Journal of Environmental Research and Public Health*.

[33] Coco A., Jacklitsch B., Williams J., Kim J.-H., Musolin K., Turner N. (2016). Criteria for a Recommended Standard: Occupational Exposure to Heat and Hot Environments. *Control Ccfd*.

[34] Casa D. J., DeMartini J. K., Bergeron M. F. (2015). National Athletic Trainers’ Association Position Statement: Exertional Heat Illnesses. *Journal of Athletic Training*.

[35] Arbury S., Lindsley M., Hodgson M. (2016). A Critical Review of OSHA Heat Enforcement Cases: Lessons Learned. *Journal of Occupational and Environmental Medicine*.

[36] Langer C. E., Mitchell D. C., Armitage T. L. (2021). Are Cal/OSHA Regulations Protecting Farmworkers in California From Heat-Related Illness?. *Journal of Occupational and Environmental Medicine*.

[37] Cheuvront S. N., Kenefick R. W., Montain S. J., Sawka M. N. (2010). Mechanisms of Aerobic Performance Impairment With Heat Stress and Dehydration. *Journal of Applied Physiology*.

[38] Hall D. M., Baumgardner K. R., Oberley T. D., Gisolfi C. V. (1999). Splanchnic Tissues Undergo Hypoxic Stress During Whole Body Hyperthermia. *American Journal of Physiology - Gastrointestinal and Liver Physiology*.

[39] Oliver S. R., Phillips N. A., Novosad V. L., Bakos M. P., Talbert E. E., Clanton T. L. (2012). Hyperthermia Induces Injury to the Intestinal Mucosa in the Mouse: Evidence for an Oxidative Stress Mechanism. *American Journal of Physiology - Regulatory, Integrative and Comparative Physiology*.

[40] Lambert G. P. (2004). Role of Gastrointestinal Permeability in Exertional Heatstroke. *Exercise and Sport Sciences Reviews*.

[41] Selkirk G. A., McLellan T. M., Wright H. E., Rhind S. G. (2008). Mild Endotoxemia, NF-Κb Translocation, and Cytokine Increase During Exertional Heat Stress in Trained and Untrained Individuals. *American Journal of Physiology - Regulatory, Integrative and Comparative Physiology*.

[42] Cui Yj, Chen Ly, Zhou X., Tang Zn, Wang C., Wang Hf (2022). Heat Stress Induced IPEC‐J2 Cells Barrier Dysfunction Through Endoplasmic Reticulum Stress Mediated Apoptosis by P‐eif2*α*/CHOP Pathway. *Journal of Cellular Physiology*.

[43] Dokladny K., Zuhl M. N., Moseley P. L. (2016). Intestinal Epithelial Barrier Function and Tight Junction Proteins With Heat and Exercise. *Journal of Applied Physiology*.

[44] McKenna Z., Houck J., Ducharme J. (2022). The Effect of Prolonged Interval and Continuous Exercise in the Heat on Circulatory Markers of Intestinal Barrier Integrity. *European Journal of Applied Physiology*.

[45] Pires W., Veneroso C. E., Wanner S. P. (2017). Association Between Exercise-Induced Hyperthermia and Intestinal Permeability: A Systematic Review. *Sports Medicine*.

[46] Ye N., Yu T., Guo H., Li J. (2019). Intestinal Injury in Heat Stroke. *Journal of Emergency Medicine*.

[47] Curi R., Lagranha C. J., Doi S. Q. (2005). Molecular Mechanisms of Glutamine Action. *Journal of Cellular Physiology*.

[48] Scalise M., Pochini L., Galluccio M., Indiveri C. (2016). Glutamine Transport. From Energy Supply to Sensing and beyond. *Biochimica et Biophysica Acta (BBA) - Bioenergetics*.

[49] Krebs H. A. (1935). Metabolism of Amino-Acids: The Synthesis of Glutamine From Glutamic Acid and Ammonia, and the Enzymic Hydrolysis of Glutamine in Animal Tissues. *Biochemical Journal*.

[50] Parry-Billings M., Newsholme E. A. (1991). The Possible Role of Glutamine Substrate Cycles in Skeletal Muscle. *Biochemical Journal*.

[51] Roth E. (2008). Nonnutritive Effects of Glutamine. *The Journal of Nutrition*.

[52] Roth E., Oehler R., Manhart N. (2002). Regulative Potential of Glutamine—Relation to Glutathione Metabolism. *Nutrition*.

[53] Blaauw R., Nel D. G., Schleicher G. K. (2020). Plasma Glutamine Levels in Relation to Intensive Care Unit Patient Outcome. *Nutrients*.

[54] Roth E., Funovics J., Mühlbacher F. (1982). Metabolic Disorders in Severe Abdominal Sepsis: Glutamine Deficiency in Skeletal Muscle. *Clinical Nutrition*.

[55] Castell L. M., Newsholme E. A. (1997). The Effects of Oral Glutamine Supplementation on Athletes After Prolonged, Exhaustive Exercise. *Nutrition*.

[56] Greig P., Grote A., Askanazi J., Michelsen C. B., Elwyn D., Kinney J. (1986). Effect of Nutritional Substrates on Muscle Amino Acid and Protein Metabolism in Surgical Patients. *Clinical Nutrition and Metabolic Research*.

[57] Souba W. W., Scott T. E., Wilmore D. W. (1985). Intestinal Consumption of Intravenously Administered Fuels. *Journal of Parenteral and Enteral Nutrition*.

[58] Brand K. (1985). Glutamine and Glucose Metabolism During Thymocyte Proliferation. Pathways of Glutamine and Glutamate Metabolism. *Biochemical Journal*.

[59] Labow B. I., Souba W. W. (2000). Glutamine. *World Journal of Surgery*.

[60] Hong R. W., Rounds J. D., Helton W. S., Robinson M. K., Wilmore D. W. (1992). Glutamine Preserves Liver Glutathione After Lethal Hepatic Injury. *Annals of Surgery*.

[61] Walsh N., Blannin A. K., Clark A. M., Cook L., Robson P., Gleeson M. (1998). The Effects of High-Intensity Intermittent Exercise on the Plasma Concentrations of Glutamine and Organic Acids. *European Journal of Applied Physiology*.

[62] Santos R. V. T., Caperuto ÉC., Costa Rosa L. F. B. P. (2007). Effects of Acute Exhaustive Physical Exercise Upon Glutamine Metabolism of Lymphocytes From Trained Rats. *Life Sciences*.

[63] Rennie M., Edwards R., Krywawych S. (1981). Effect of Exercise on Protein Turnover in Man. *Clinical Science*.

[64] Parry-Billings M., Budgett R., Koutedakis Y (1992). Plasma Amino Acid Concentrations in the Overtraining Syndrome: Possible Effects on the Immune System. *Medicine & Science in Sports & Exercise*.

[65] Van Hall G., Saris W., Wagenmakers A. (1998). Effect of Carbohydrate Supplementation on Plasma Glutamine During Prolonged Exercise and Recovery. *International Journal of Sports Medicine*.

[66] Kargotich S., Rowbottom D. G., Keast D., Goodman C., Dawson B., Morton A. R. (2005). Plasma Glutamine Changes After High-Intensity Exercise in Elite Male Swimmers. *Research in Sports Medicine*.

[67] Krishna M. G., Coker R. H., Lacy D. B., Zinker B. A., Halseth A. E., Wasserman D. H. (2000). Glucagon Response to Exercise Is Critical for Accelerated Hepatic Glutamine Metabolism and Nitrogen Disposal. *American Journal of Physiology - Endocrinology And Metabolism*.

[68] Halseth A. E., Rhéaume N., Messina A. B. (1998). Regulation of Hepatic Glutamine Metabolism During Exercise in the Dog. *American Journal of Physiology - Endocrinology And Metabolism*.

[69] Wasserman D. H., Geer R. J., Williams P. E., Becker T., Lacy D. B., Abumrad N. N. (1991). Interaction of Gut and Liver in Nitrogen Metabolism During Exercise. *Metabolism*.

[70] Chaudhry F. A., Reimer R. J., Edwards R. H. (2002). The Glutamine Commute: Take the N Line and Transfer to the A. *The Journal of Cell Biology*.

[71] Stumvoll M., Perriello G., Meyer C., Gerich J. (1999). Role of Glutamine in Human Carbohydrate Metabolism in Kidney and Other Tissues. *Kidney International*.

[72] Schemitt E. G., Hartmann R. M., Colares J. R. (2019). Protective Action of Glutamine in Rats With Severe Acute Liver Failure. *World Journal of Hepatology*.

[73] Cruzat V. F., Tirapegui J. (2009). Effects of Oral Supplementation With Glutamine and Alanyl-Glutamine on Glutamine, Glutamate, and Glutathione Status in Trained Rats and Subjected to Long-Duration Exercise. *Nutrition*.

[74] Windmueller H. G., Spaeth A. E. (1974). Uptake and Metabolism of Plasma Glutamine by the Small Intestine. *Journal of Biological Chemistry*.

[75] Newsholme E., Carrie A. (1994). Quantitative Aspects of Glucose and Glutamine Metabolism by Intestinal Cells. *Gut*.

[76] Kim M.-H., Kim H. (2017). The Roles of Glutamine in the Intestine and Its Implication in Intestinal Diseases. *International Journal of Molecular Sciences*.

[77] Rao R., Samak G. (2012). Role of Glutamine in Protection of Intestinal Epithelial Tight Junctions. *Journal of Epithelial Biology & Pharmacology*.

[78] Dechelotte P., Darmaun D., Rongier M., Hecketsweiler B., Rigal O., Desjeux J.-F. (1991). Absorption and Metabolic Effects of Enterally Administered Glutamine in Humans. *American Journal of Physiology - Gastrointestinal and Liver Physiology*.

[79] Fleming S. E., Zambell K. L., Fitch M. D. (1997). Glucose and Glutamine Provide Similar Proportions of Energy to Mucosal Cells of Rat Small Intestine. *American Journal of Physiology - Gastrointestinal and Liver Physiology*.

[80] Souba W. W., Herskowitz K., Klimberg V. S. (1990). The Effects of Sepsis and Endotoxemia on Gut Glutamine Metabolism. *Annals of Surgery*.

[81] Mallet R. T., Kelleher J. K., Jackson M. J. (1986). Substrate Metabolism of Isolated Jejunal Epithelium: Conservation of Three-Carbon Units. *American Journal of Physiology: Cell Physiology*.

[82] Calder P., Yaqoob P. (1999). Glutamine and the Immune System. *Amino Acids*.

[83] Curi T. C. P., De Melo M. P., De Azevedo R. B., Zorn T. M., Curi R. (1997). Glutamine Utilization by Rat Neutrophils: Presence of Phosphate-dependent Glutaminase. *American Journal of Physiology: Cell Physiology*.

[84] Feng X., Li X., Liu N., Hou N., Sun X., Liu Y. (2022). Glutaminolysis and CD4+ T-Cell Metabolism in Autoimmunity: FROM Pathogenesis to Therapy Prospects. *Frontiers in Immunology*.

[85] Liu J.-Q., Geng X.-R., Hu T.-Y. (2022). Glutaminolysis Is Required in Maintaining Immune Regulatory Functions in B Cells. *Mucosal Immunology*.

[86] Curi R., Lagranha C., Doi S., Sellitti D., Procopio J., Pithon‐Curi T. (2005). Glutamine‐dependent Changes in Gene Expression and Protein Activity. *Cell Biochemistry and Function*.

[87] Garcia C., Pithon-Curi T. C., De Lourdes Firmano M., Pires De Melo M., Newsholme P., Curi R. (1999). Effects of Adrenaline on Glucose and Glutamine Metabolism and Superoxide Production by Rat Neutrophils. *Clinical Science*.

[88] Sadaf A., Quinn C. T. (2020). L-Glutamine for Sickle Cell Disease: Knight or Pawn?. *Experimental Biology and Medicine*.

[89] Zuhl M., Dokladny K., Mermier C., Schneider S., Salgado R., Moseley P. (2015). The Effects of Acute Oral Glutamine Supplementation on Exercise-Induced Gastrointestinal Permeability and Heat Shock Protein Expression in Peripheral Blood Mononuclear Cells. *Cell Stress & Chaperones*.

[90] Pugh J. N., Sage S., Hutson M. (2017). Glutamine Supplementation Reduces Markers of Intestinal Permeability During Running in the Heat in a Dose-Dependent Manner. *European Journal of Applied Physiology*.

[91] Nava R. C., Zuhl M. N., Moriarty T. A (2019). The Effect of Acute Glutamine Supplementation on Markers of Inflammation and Fatigue During Consecutive Days of Simulated Wildland Firefighting. *Journal of Occupational and Environmental Medicine*.

[92] Moore M., Moriarty T. A., Connolly G. (2019). Oral Glutamine Supplement Reduces Subjective Fatigue Ratings During Repeated Bouts of Firefighting Simulations. *Safety Now*.

[93] Chow A., Zhang R. (1998). Glutamine Reduces Heat Shock–Induced Cell Death in Rat Intestinal Epithelial Cells. *The Journal of Nutrition*.

[94] Wu Q. J., Jiao C., Liu Z. H. (2021). Effect of Glutamine on the Growth Performance, Digestive Enzyme Activity, Absorption Function, and mRNA Expression of Intestinal Transporters in Heat-Stressed Chickens. *Research in Veterinary Science*.

[95] Zhang B., Sun H., Sun Z., Liu N., Liu R., Zhong Q. (2023). Glutamine Alleviated Heat Stress-Induced Damage of Porcine Intestinal Epithelium Associated With the Mitochondrial Apoptosis Pathway Mediated by Heat Shock Protein 70. *Journal of Animal Science*.

[96] Soares A. D., Costa K. A., Wanner S. P. (2014). Dietary Glutamine Prevents the Loss of Intestinal Barrier Function and Attenuates the Increase in Core Body Temperature Induced by Acute Heat Exposure. *The British Journal of Nutrition*.

[97] Singleton K. D., Wischmeyer P. E. (2006). Oral Glutamine Enhances Heat Shock Protein Expression and Improves Survival Following Hyperthermia. *Shock*.

[98] Kallweit A. R., Baird C. H., Stutzman D. K., Wischmeyer P. E. (2012). Glutamine Prevents Apoptosis in Intestinal Epithelial Cells and Induces Differential Protective Pathways in Heat and Oxidant Injury Models. *Journal of Parenteral and Enteral Nutrition*.

[99] Wischmeyer P. E., Musch M. W., Madonna M. B., Thisted R., Chang E. B. (1997). Glutamine Protects Intestinal Epithelial Cells: Role of Inducible HSP70. *American Journal of Physiology—Gastrointestinal and Liver Physiology*.

[100] Osborne J. O., Stewart I. B., Beagley K. W., Borg D. N., Minett G. M. (2019). Acute Glutamine Supplementation Does Not Improve 20-km Self-Paced Cycling Performance in the Heat. *European Journal of Applied Physiology*.

[101] Ogden H. B., Fallowfield J. L., Child R. B. (2022). No Protective Benefits of Low Dose Acute L-Glutamine Supplementation on Small Intestinal Permeability, Epithelial Injury and Bacterial Translocation Biomarkers in Response to Subclinical Exertional-Heat Stress: A Randomized Cross-Over Trial. *Temperature*.

[102] Ogden H. B., Fallowfield J. L., Child R. B. (2022). Acute L-Glutamine Supplementation Does Not Improve Gastrointestinal Permeability, Injury or Microbial Translocation in Response to Exhaustive High Intensity Exertional-Heat Stress. *European Journal of Sport Science*.

[103] Phanvijhitsiri K., Musch M. W., Ropeleski M. J., Chang E. B. (2006). Heat Induction of Heat Shock Protein 25 Requires Cellular Glutamine in Intestinal Epithelial Cells. *American Journal of Physiology: Cell Physiology*.

[104] Wong H. R., Ryan M., Wispé J. R. (1997). The Heat Shock Response Inhibits Inducible Nitric Oxide Synthase Gene Expression by Blocking I*κ*-B Degradation and NF-Κb Nuclear Translocation. *Biochemical and Biophysical Research Communications*.

[105] Morimoto R. I., Santoro M. G. (1998). Stress–Inducible Responses and Heat Shock Proteins: New Pharmacologic Targets for Cytoprotection. *Nature Biotechnology*.

[106] Rakoff-Nahoum S., Paglino J., Eslami-Varzaneh F., Edberg S., Medzhitov R. (2004). Recognition of Commensal Microflora by Toll-Like Receptors Is Required for Intestinal Homeostasis. *Cell*.

[107] Hu S., Ciancio M. J., Lahav M. (2007). Translational Inhibition of Colonic Epithelial Heat Shock Proteins by IFN-*γ* and TNF-*α* in Intestinal Inflammation. *Gastroenterology*.

[108] Hamiel C. R., Pinto S., Hau A., Wischmeyer P. E. (2009). Glutamine Enhances Heat Shock Protein 70 Expression via Increased Hexosamine Biosynthetic Pathway Activity. *American Journal of Physiology: Cell Physiology*.

[109] Kazemi Z., Chang H., Haserodt S., McKen C., Zachara N. E. (2010). O-Linked *β*-N-Acetylglucosamine (O-GlcNAc) Regulates Stress-Induced Heat Shock Protein Expression in a GSK-3*β*-Dependent Manner. *Journal of Biological Chemistry*.

[110] Zhang Y., Huang L., Zhang J., Moskophidis D., Mivechi N. F. (2002). Targeted Disruption of Hsf1 Leads to Lack of Thermotolerance and Defines Tissue‐Specific Regulation for Stress‐Inducible Hsp Molecular Chaperones. *Journal of Cellular Biochemistry*.

[111] Gong J., Jing L. (2011). Glutamine Induces Heat Shock Protein 70 Expression via O-GlcNAc Modification and Subsequent Increased Expression and Transcriptional Activity of Heat Shock Factor-1. *Minerva Anestesiologica*.

[112] Dokladny K., Moseley P. L., Ma T. Y. (2006). Physiologically Relevant Increase in Temperature Causes an Increase in Intestinal Epithelial Tight Junction Permeability. *American Journal of Physiology—Gastrointestinal and Liver Physiology*.

[113] Akbari P., Braber S., Alizadeh A. (2015). Galacto-Oligosaccharides Protect the Intestinal Barrier by Maintaining the Tight Junction Network and Modulating the Inflammatory Responses after a Challenge With the Mycotoxin Deoxynivalenol in Human Caco-2 Cell Monolayers and B6C3F1 Mice. *The Journal of Nutrition*.

[114] Xia Z., Huang L., Yin P. (2019). L-Arginine Alleviates Heat Stress-Induced Intestinal Epithelial Barrier Damage by Promoting Expression of Tight Junction Proteins via the AMPK Pathway. *Molecular Biology Reports*.

[115] Kamm K. E., Stull J. T. (2001). Dedicated Myosin Light Chain Kinases With Diverse Cellular Functions. *Journal of Biological Chemistry*.

[116] Yang P. C., He S. H., Zheng P. Y. (2007). Investigation Into the Signal Transduction Pathway via Which Heat Stress Impairs Intestinal Epithelial Barrier Function. *Journal of Gastroenterology and Hepatology*.

[117] Wang B., Wu Z., Ji Y., Sun K., Dai Z., Wu G. (2016). L-Glutamine Enhances Tight Junction Integrity by Activating CaMK Kinase 2–AMP-Activated Protein Kinase Signaling in Intestinal Porcine Epithelial Cells. *The Journal of Nutrition*.

[118] Seger R., Krebs E. G. (1995). The MAPK Signaling Cascade. *The FASEB Journal*.

[119] Larson S. D., Li J., Chung D. H., Evers B. M. (2007). Molecular Mechanisms Contributing to Glutamine-Mediated Intestinal Cell Survival. *American Journal of Physiology—Gastrointestinal and Liver Physiology*.

[120] Hou Y.-C., Chiu W.-C., Yeh C.-L., Yeh S.-L. (2012). Glutamine Modulates Lipopolysaccharide-Induced Activation of NF-Κb via the Akt/mTOR Pathway in Lung Epithelial Cells. *American Journal of Physiology—Lung Cellular and Molecular Physiology*.

[121] Karin M., Ben-Neriah Y. (2000). Phosphorylation Meets Ubiquitination: The Control of NF-Κb Activity. *Annual Review of Immunology*.

[122] Afonina I. S., Zhong Z., Karin M., Beyaert R. (2017). Limiting Inflammation—The Negative Regulation of NF-Κb and the NLRP3 Inflammasome. *Nature Immunology*.

[123] Pasparakis M. (2009). Regulation of Tissue Homeostasis by NF-Κb Signalling: Implications for Inflammatory Diseases. *Nature Reviews Immunology*.

[124] Baldwin A. S. (1996). The NF-Κb and I*κ*B Proteins: New Discoveries and Insights. *Annual Review of Immunology*.

[125] Barnes P. J., Karin M. (1997). Nuclear Factor-Κb—A Pivotal Transcription Factor in Chronic Inflammatory Diseases. *New England Journal of Medicine*.

[126] Liu T., Zhang L., Joo D., Sun S.-C. (2017). NF-*κ*B Signaling in Inflammation. *Signal Transduction and Targeted Therapy*.

[127] Baltimore D. (2011). NF-*κ*B Is 25. *Nature Immunology*.

[128] Guma M., Stepniak D., Shaked H. (2011). Constitutive Intestinal NF-Κb Does Not Trigger Destructive Inflammation Unless Accompanied by MAPK Activation. *Journal of Experimental Medicine*.

[129] Patra A. K., Kar I. (2021). Heat Stress on Microbiota Composition, Barrier Integrity, and Nutrient Transport in Gut, Production Performance, and Its Amelioration in Farm Animals. *Journal of Animal Science And Technology*.

[130] Xia Z.-N., Zong Y., Zhang Z.-T. (2017). Dexmedetomidine Protects Against Multi-Organ Dysfunction Induced by Heatstroke via Sustaining the Intestinal Integrity. *Shock*.

[131] Xiong Y., Cao S., Xiao H. (2022). Alterations in Intestinal Microbiota Composition Coincide With Impaired Intestinal Morphology and Dysfunctional Ileal Immune Response in Growing-Finishing Pigs Under Constant Chronic Heat Stress. *Journal of Animal Science and Biotechnology*.

[132] Brasse-Lagnel C. G., Lavoinne A. M., Husson A. S. (2010). Amino Acid Regulation of Mammalian Gene Expression in the Intestine. *Biochimie*.

[133] Lecleire S., Hassan A., Marion-Letellier R. (2008). Combined Glutamine and Arginine Decrease Proinflammatory Cytokine Production by Biopsies From Crohn’s Patients in Association With Changes in Nuclear Factor-Κb and P38 Mitogen-Activated Protein Kinase Pathways3. *The Journal of Nutrition*.

[134] Hubert-Buron A., Leblond J., Jacquot A., Ducrotté P., Déchelotte P., Coëffier M. (2006). Glutamine Pretreatment Reduces IL-8 Production in Human Intestinal Epithelial Cells by Limiting I*κ*B*α* Ubiquitination. *The Journal of Nutrition*.

[135] Dokladny K., Lobb R., Wharton W., Ma T. Y., Moseley P. L. (2010). LPS-Induced Cytokine Levels Are Repressed by Elevated Expression of HSP70 in Rats: Possible Role of NF-*κ*B. *Cell Stress & Chaperones*.

[136] Hall D. M., Buettner G. R., Oberley L. W., Xu L., Matthes R. D., Gisolfi C. V. (2001). Mechanisms of Circulatory and Intestinal Barrier Dysfunction During Whole Body Hyperthermia. *American Journal of Physiology - Heart and Circulatory Physiology*.

[137] Yu J., Liu F., Yin P. (2013). Involvement of Oxidative Stress and Mitogen-Activated Protein Kinase Signaling Pathways in Heat Stress-Induced Injury in the Rat Small Intestine. *Stress: The International Journal on the Biology of Stress*.

[138] Rouse K., Nwokedi E., Woodliff J. E., Epstein J., Klimberg V. S. (1995). Glutamine Enhances Selectivity of Chemotherapy Through Changes in Glutathione Metabolism. *Annals of Surgery*.

[139] Cao Y., Feng Z., Hoos A., Klimberg V. S. (1998). Glutamine Enhances Gut Glutathione Production. *Journal of Parenteral and Enteral Nutrition*.

[140] Harward T. R., Coe D., Souba W. W., Klingman N., Seeger J. M. (1994). Glutamine Preserves Gut Glutathione Levels During Intestinal Ischemia/reperfusion. *Journal of Surgical Research*.

[141] Patlevič P., Vašková J., Švorc P., Vaško L., Švorc P. (2016). Reactive Oxygen Species and Antioxidant Defense in Human Gastrointestinal Diseases. *Integrative Medicine Research*.

[142] Bouchama A., Al-Sedairy S., Siddiqui S., Shail E., Bezeig M. (1993). Elevated Pyrogenic Cytokines in Heatstroke. *Chest*.

[143] Hashim I., Al-Zeer A., Al-Shohaib S., Al-Ahwal M., Shenkin A. (1997). Cytokine Changes in Patients With Heatstroke During Pilgrimage to Makkah. *Mediators of Inflammation*.

[144] Seckinger P., Isaaz S., Dayer J. (1989). Purification and Biologic Characterization of a Specific Tumor Necrosis Factor *α* Inhibitor. *Journal of Biological Chemistry*.

[145] Hannum C. H., Wilcox C. J., Arend W. P. (1990). Interleukin-1 Receptor Antagonist Activity of a Human Interleukin-1 Inhibitor. *Nature*.

[146] Bouchama A., Mohanna F. A., El-Sayed R. (2005). Experimental Heatstroke in Baboon: Analysis of the Systemic Inflammatory Response. *Shock*.

[147] Leon L. R. (2006). The Thermoregulatory Consequences of Heat Stroke: Are Cytokines Involved?. *Journal of Thermal Biology*.

[148] Du L.-W., Xu B.-Q., Xun K., Zhang F.-Q. (2023). Glutamine Supplementation Attenuates Intestinal Apoptosis by Inducing Heat Shock Protein 70 in Heatstroke Rats. *World Journal of Emergency Medicine*.

[149] Hailes W. S., Slivka D., Cuddy J., Ruby B. C. (2011). Human Plasma Inflammatory Response During 5 Days of Exercise Training in the Heat. *Journal of Thermal Biology*.

[150] Barberio M., Elmer D., Laird R., Lee K., Gladden B., Pascoe D. (2014). Systemic LPS and Inflammatory Response During Consecutive Days of Exercise in Heat. *International Journal of Sports Medicine*.

[151] Zheng C., Chen X.-K., Zhou Y. (2018). Acute Glutamine Ingestion Modulates Lymphocytic Responses to Exhaustive Exercise in the Heat. *Applied Physiology Nutrition and Metabolism*.

[152] Yaqoob P., Calder P. C. (1997). Glutamine Requirement of Proliferating T Lymphocytes. *Nutrition*.

[153] Hirakawa R., Nurjanah S., Furukawa K. (2020). Heat Stress Causes Immune Abnormalities via Massive Damage to Effect Proliferation and Differentiation of Lymphocytes in Broiler Chickens. *Frontiers in Veterinary Science*.

[154] Dokladny K., Zuhl M. N., Mandell M. (2013). Regulatory Coordination Between Two Major Intracellular Homeostatic Systems: Heat Shock Response and Autophagy. *Journal of Biological Chemistry*.

[155] Kempaiah P., Dokladny K., Karim Z. (2016). Reduced Hsp70 and Glutamine in Pediatric Severe Malaria Anemia: Role of Hemozoin in Suppressing Hsp70 and NF-*κ*B Activation. *Molecular Medicine*.

[156] Slimen I. B., Najar T., Ghram A., Dabbebi H., Ben Mrad M., Abdrabbah M. (2014). Reactive Oxygen Species, Heat Stress and Oxidative-Induced Mitochondrial Damage. A Review. *International Journal of Hyperthermia*.

[157] Turrens J. F. (2003). Mitochondrial Formation of Reactive Oxygen Species. *The Journal of Physiology*.

[158] Hillman A. R., Vince R. V., Taylor L., McNaughton L., Mitchell N., Siegler J. (2011). Exercise-induced Dehydration With and Without Environmental Heat Stress Results in Increased Oxidative Stress. *Applied Physiology Nutrition and Metabolism*.

[159] Ncho C. M., Gupta V., Choi Y.-H. (2023). Effects of Dietary Glutamine Supplementation on Heat-Induced Oxidative Stress in Broiler Chickens: A Systematic Review and Meta-Analysis. *Antioxidants*.

[160] Silvestrini A., Meucci E., Ricerca B. M., Mancini A. (2023). Total Antioxidant Capacity: Biochemical Aspects and Clinical Significance. *International Journal of Molecular Sciences*.

[161] Wittbrodt M. T., Millard-Stafford M. (2018). Dehydration Impairs Cognitive Performance: A Meta-Analysis. *Medicine & Science in Sports & Exercise*.

[162] Goulet E. D. (2013). Effect of Exercise-Induced Dehydration on Endurance Performance: Evaluating the Impact of Exercise Protocols on Outcomes Using a Meta-Analytic Procedure. *British Journal of Sports Medicine*.

[163] Godek S. F., Peduzzi C., Burkholder R., Condon S., Dorshimer G., Bartolozzi A. R. (2010). Sweat Rates, Sweat Sodium Concentrations, and Sodium Losses in 3 Groups of Professional Football Players. *Journal of Athletic Training*.

[164] Bates G. P., Miller V. S. (2008). Sweat Rate and Sodium Loss During Work in the Heat. *Journal of Occupational Medicine and Toxicology*.

[165] Ray M. L., Bryan M. W., Ruden T. M., Baier S. M., Sharp R. L., King D. S. (1998). Effect of Sodium in a Rehydration Beverage When Consumed as a Fluid or Meal. *Journal of Applied Physiology*.

[166] Buccigrossi V., Lo Vecchio A., Bruzzese E. (2020). Potency of Oral Rehydration Solution in Inducing Fluid Absorption Is Related to Glucose Concentration. *Scientific Reports*.

[167] Wright E. M., Loo D. D. (2000). Coupling Between Na+, Sugar, and Water Transport Across the Intestine. *Annals of the New York Academy of Sciences*.

[168] Coëffier M., Hecketsweiler B., Hecketsweiler P., Déchelotte P. (2005). Effect of Glutamine on Water and Sodium Absorption in Human Jejunum at Baseline and During PGE1-Induced Secretion. *Journal of Applied Physiology*.

[169] Hoffman J. R., Ratamess N. A., Kang J. (2010). Examination of the Efficacy of Acute L-Alanyl-L-Glutamine Ingestion During Hydration Stress in Endurance Exercise. *Journal of the International Society of Sports Nutrition*.

[170] Pruna G. J., Hoffman J. R., McCormack W. P. (2016). Effect of Acute L-Alanyl-L-Glutamine and Electrolyte Ingestion on Cognitive Function and Reaction Time Following Endurance Exercise. *European Journal of Sport Science*.

[171] Amann M. (2011). Central and Peripheral Fatigue: Interaction During Cycling Exercise in Humans. *Medicine & Science in Sports & Exercise*.

[172] Finsterer J. (2012). Biomarkers of Peripheral Muscle Fatigue During Exercise. *BMC Musculoskeletal Disorders*.

[173] Meeusen R., Watson P., Hasegawa H., Roelands B., Piacentini M. F. (2006). Central Fatigue: the Serotonin Hypothesis and beyond. *Sports Medicine*.

[174] Ando S., Kimura T., Hamada T., Kokubu M., Moritani T., Oda S. (2005). Increase in Reaction Time for the Peripheral Visual Field During Exercise Above the Ventilatory Threshold. *European Journal of Applied Physiology*.

[175] O’Neill C., Panuwatwanich K. The Impact of Fatigue on Labour Productivity: Case Study of Dam Construction Project in Queensland.

[176] Coqueiro A. Y., Rogero M. M., Tirapegui J. (2019). Glutamine as an Anti-Fatigue Amino Acid in Sports Nutrition. *Nutrients*.

[177] Bassini-Cameron A., Monteiro A., Gomes A., Werneck-de-Castro J., Cameron L. (2008). Glutamine Protects Against Increases in Blood Ammonia in Football Players in an Exercise Intensity-Dependent Way. *British Journal of Sports Medicine*.

[178] Xiang J., Ennis S. R., Abdelkarim G. E., Fujisawa M., Kawai N., Keep R. F. (2003). Glutamine Transport at the Blood–Brain and Blood–Cerebrospinal Fluid Barriers. *Neurochemistry International*.

[179] Gareau M. G. (2014). Microbiota-Gut-Brain Axis and Cognitive Function. *Microbial Endocrinology: The Microbiota-Gut-Brain Axis in Health and Disease*.

[180] Dos Santos Quaresma M., Souza W., Lemos V., Caris A., Thomatieli-Santos R. (2020). The Possible Importance of Glutamine Supplementation to Mood and Cognition in Hypoxia From High Altitude. *Nutrients*.

[181] Krieger J. W., Crowe M., Blank S. E. (2004). Chronic Glutamine Supplementation Increases Nasal but Not Salivary IgA During 9 Days of Interval Training. *Journal of Applied Physiology*.

[182] Parsons L. A., Shindell D., Tigchelaar M., Zhang Y., Spector J. T. (2021). Increased Labor Losses and Decreased Adaptation Potential in a Warmer World. *Nature Communications*.

